# The Immunological Conundrum of Endogenous Retroelements

**DOI:** 10.1146/annurev-immunol-101721-033341

**Published:** 2023-01-11

**Authors:** George Kassiotis

**Affiliations:** 1 Retroviral Immunology Laboratory, The Francis Crick Institute, London, United Kingdom; 2 Department of Infectious Disease, Faculty of Medicine, Imperial College London, London, United Kingdom

**Keywords:** endogenous retrovirus, endogenous retroelement, interferon response, adaptive immunity

## Abstract

Our defenses against infection rely on the ability of the immune system to distinguish invading pathogens from self. This task is exceptionally challenging, if not seemingly impossible, in the case of retroviruses that have integrated almost seamlessly into the host. This review examines the limits of innate and adaptive immune responses elicited by endogenous retroviruses and other retroelements, the targets of immune recognition, and the consequences for host health and disease. Contrary to theoretical expectation, endogenous retroelements retain substantial immunogenicity, which manifests most profoundly when their epigenetic repression is compromised, contributing to autoinflammatory and autoimmune disease and age-related inflammation. Nevertheless, recent evidence suggests that regulated immune reactivity to endogenous retroelements is integral to immune system development and function, underpinning cancer immunosurveillance, resistance to infection, and responses to the microbiota. Elucidation of the interaction points with endogenous retroelements will therefore deepen our understanding of immune system function and contribution to disease.

## Introduction

To mount an appropriate response against infectious pathogens, the immune system must gauge the threat each pathogen poses, by analyzing its molecular components and their dissimilarity to the host. This task is hindered by enormous variability in pathogen persistence and immunogenicity (1). These two properties are intricately linked, as pathogen persistence in the host requires evasion of host immunity. Consequently, many pathogens have evolved mechanisms for stealth or active suppression of the immune response (1). Thereby, life-long infection can be achieved, in spite of or at an equilibrium with host immunity. An example of this strategy is mimicry, whereby pathogen components molecularly or functionally resemble the host.^1^ With all their components made by the host cell, viruses are particularly successful at mimicry, which they use at multiple levels.

Molecular mimicry has long been proposed as a strategy that allows persistence of pathogens whose antigens are similar to host antigens by exploiting self-tolerance (2). However, as self-tolerance may be incomplete, molecular mimicry of T cell and B cell antigens is also a potential trigger of autoimmunity (3). Viral mimicry of key host immune mediators also allows viruses to suppress or subvert host immunity, and several viruses express functional host protein mimics, such as cytokines, chemokines, and their receptors (4), or antagonists of antiviral host signaling cascades, such as the interferon response (5, 6).

Blurring the boundaries between virus and host is an evolutionary strategy of many virus families establishing chronic infection, but none has taken it to the extreme form that retroviruses have. By virtue of their ability to integrate functional copies of their genome into the host germline DNA, they can become a true part of the host. Indeed, our DNA is host to hundreds of thousands of retrovirus integrations, acquired over successive waves of retrovirus infection and further amplification in the germline. It is also host to numerous other types of retrotransposable elements, collectively referred to here as endogenous retroelements (EREs): genomic parasites that also rely on reverse transcription and integration of their genomes into the host DNA.

Although the vast majority of ERE integrations are mutated, incomplete, or defective genomic copies, many have retained the ability to complete some or all of the steps in the retroelement replication cycle, including transcription, translation or reverse transcription, and reintegration. Retention of this ability, combined with the sheer number of EREs in the genome, provides an extensive interface of interaction with host physiology and pathology. The influence of regulatory sequences provided by EREs on host gene function has long been recognized (7, 8), and immune genes are no exception. The full extent to which EREs shape immune gene function and evolution is likely underestimated owing to the incomplete annotation of ERE transcripts, but appreciation is growing and comprehensive efforts are underway; these are not the focus of this review.

This extreme form of parasitism—where EREs become part of self—may be considered the endgame for host immunity against them. Nevertheless, both innate and adaptive host immune responses against EREs are observed, and the supporting evidence is reviewed here. Also reviewed is the potential association of retained ERE immunogenicity not only with the control of EREs themselves, but also with overall immune reactivity to foreign or other self-targets, whereby they might tune resistance to infection, equilibrium with commensals, immunosurveillance of cancer, or development of autoimmunity.

## Endogenous Retroelement Life Cycle and Evolution

With nucleic acids being the cornerstone of all life, it is perhaps expected that genetic material will be exchanged between exogenous viruses, EREs, and their hosts, providing the substrate for coevolution. The focus in this review is on EREs (class I transposable elements), defined as transposable elements with reverse transcription and host germline integration of their genomes as obligatory steps in their replication cycle (9). This definition excludes DNA (class II) transposable elements, which lack an RNA intermediate (9). It also excludes endogenous Borna-like N(EBLN) elements that are fixed in the human population (10) and human herpesvirus 6 copies, which are found in the germline of ~1% of humans, the integration of which was likely accidental.

**Figure 1:**
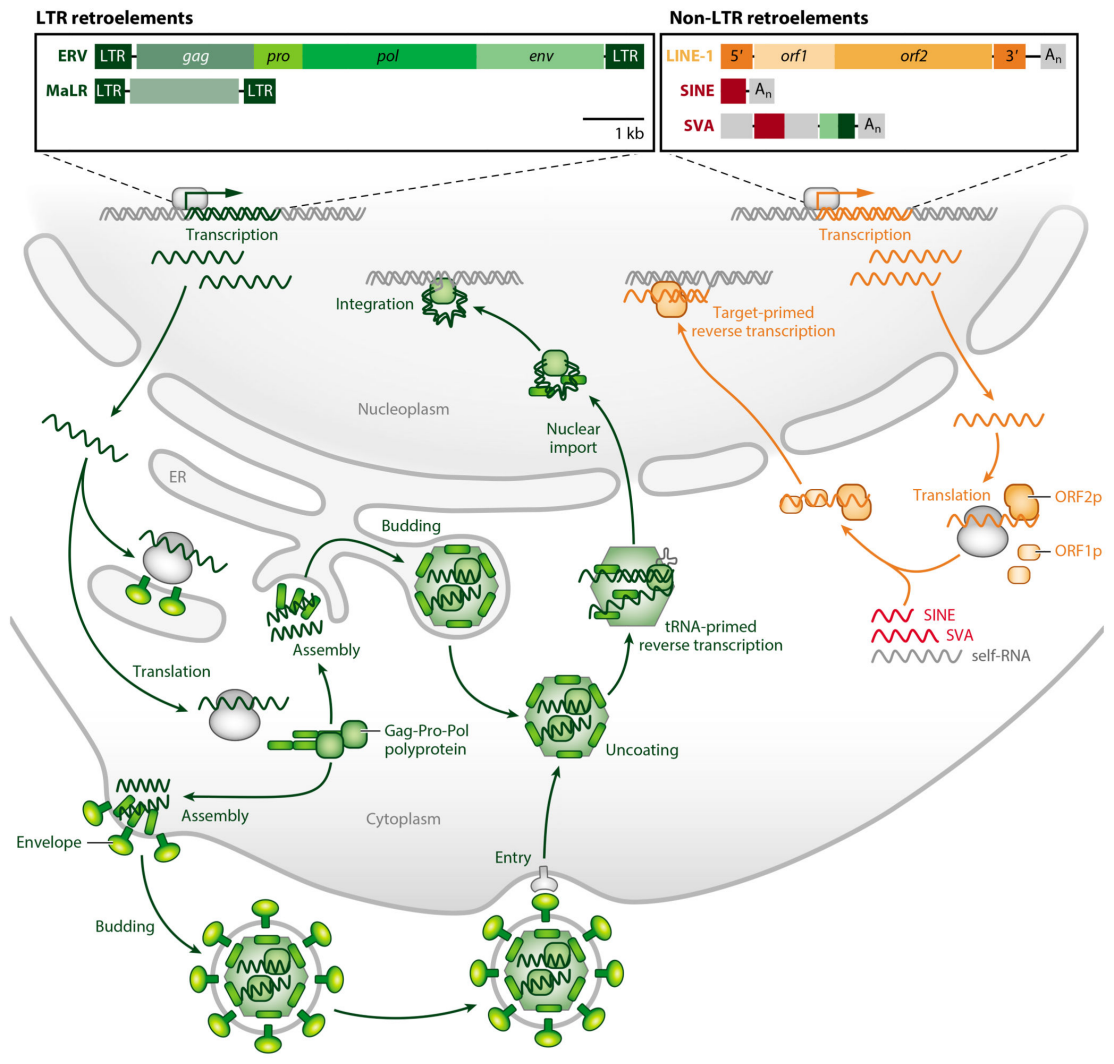
Genome structure and replication cycles of LTR (*left*) and non-LTR retroelements (*right*). Genomes of canonical LTR retroelements, including ERV and MaLR (*green*), and of non-LTR retroelements, including LINE-1 (*orange*), SINE, and SVA (*red*). SVAs are composite repetitive elements comprising a CCCTCT repeat section (*first gray rectangle*), two *Alu-like* sequences in reverse orientation, a VNTR section (*second gray rectangle*), and a region derived from ERV *env* and 5′ LTR. Also depicted are the *env* (envelope), *gag* (group-specific antigen), and *pro-pol* (protease-polymerase) ORFs of the complete ERV and the *orf1* and *orf2* ORFs of the complete LINE-1. Also depicted are the poly-A regions (A_n_) at the 3′ end of non-LTR elements. Not depicted are accessory ORFs in more complex ERVs [e.g., *rec* and *np9* in certain HERV-K(HML2) proviruses] or the *orf0* present in reverse orientation in the 5′ UTR of LINE-1. Transcribed ERV RNA is exported to the cytoplasm, where it serves as mRNA for translation of functional proteins or as genomic RNA. Assembly of ERV particles typically takes place at the plasma membrane, with budding into the extracellular space, but ERVs lacking *env* and MaLR elements have adopted intracellular budding, such as into the ER or other subcellular locations. Reverse transcription of ERV genomic RNA is primed by host tRNA in the uncoated virus particle core and the pre-integration complex is then imported into the nucleus for integration of the virus DNA into the host DNA. Transcribed LINE-1 RNA is also exported to the cytoplasm acting as mRNA and genomic RNA, with the translated proteins, particularly ORF2p, which is made in limited amounts, exhibiting *cis* preference for the LINE-1 mRNA that has produced it. Nevertheless, SINE or SVA RNA, and, in principle, any polyadenylated host RNA (self-RNA), may also bind ORF2p. The complexes are imported into the nucleus, where ORF2p attacks host DNA, which is used to prime reverse transcription of associated RNA and integration of the complementary DNA (cDNA) copy. Abbreviations: ER, endoplasmic reticulum; ERV, endogenous retrovirus; LINE-1, long interspersed nuclear elements 1; LTR, long terminal repeat; MaLR, mammalian apparent LTR retrotransposon; ORF, open reading frame; SINE, short interspersed nuclear element; SVA, SINE-VNTR-*Alu*; UTR, untranslated region; VNTR, variable-number tandem repeat.

EREs in the human genome comprise phylogenetically diverse families, but a major distinction relates to their genomic structure and presumed origin (Figure 1). Deriving from germline infection by exogenous retroviruses, endogenous retroviruses (ERVs) exhibit the typical structure of retroviral genomes with *gag*, *pro-pol*, and *env* open reading frames (ORFs) flanked by directly repeated long terminal repeats (LTRs). Whereas *gag*, encoding capsid proteins, and *pro-pol*, encoding enzymatic activities, are necessary for autonomous replication of ERVs, *env*, encoding the envelope glycoprotein that mediates entry, is necessary only for infection of new target cells. Consequently, once in the germline, even ERVs that have lost the *env* ORF can still amplify their copies, sometimes more successfully, by retrotransposition (11).

The ability of ERVs to complete the replication cycle is ultimately disrupted by accumulated mutation or loss of ORFs or LTR sequences over evolutionary time. Indeed, the vast majority of ERVs in mammalian genomes are replication defective, with only the most recently acquired copies retaining functional ORFs or replication capacity, which varies between species. This also varies within species, as recent ERV integrations may also exhibit insertional polymorphism between individuals.

Likely owing to selection of phenotypic traits, such as cancer susceptibility, caused by ERV insertional mutagenesis during their establishment, laboratory mouse strains carry an unusually high burden of complete or near-complete ERVs (12). Germline integrations of replication-competent mouse mammary tumor viruses (MMTVs) and murine leukemia viruses (MLVs) are present in several laboratory mouse strains, able to transmit as both endogenous and exogenous retroviruses (13). This duality confounded early investigations into the genetics of cancer but ultimately led to the discovery of ERVs (12). Laboratory mouse strains lacking endogenous MLVs that are able to replicate in murine cells still carry multiple related defective integrations with most ORFs still intact. When expressed in the same cell, these defective copies collectively produce all the components required to form a transducing retrovirus particle, with each functional component donated by a different provirus. Such trans-complementation permits the mobilization of individually defective endogenous MLVs, generating new integrations. Moreover, recombination between defective endogenous MLV genomes during such mobilization can also restore the replication defects, giving rise to fully infectious MLVs, transmitted as exogenous retroviruses. This process is exemplified by the restoration of infectivity, through recombination, of *Emv2*, a single-copy defective endogenous MLV with an ecotropic *env* in commonly used C57BL/6 mice (14–18). Infectious *Emv2* recombinant MLVs have been discovered in murine cancer cell lines, as well as in immunodeficient mouse strains, highlighting the relatively high frequency of infectivity restoration (14–18).

Human ERVs (HERVs) are thought to be generally older and, therefore, more defective than murine ERVs. Consequently, no HERV is thought to have retained full replication capacity. Nevertheless, proviruses with seemingly intact ORFs are found within the HERV-K(HML-2) family (19). The human MMTV-like (HML) families of HERV-K share similarities with MMTVs, as the name suggests, but represent diverse groups of proviruses that entered our ancestors’ germlines at different times during evolution (19). The HERV-K(HML-2) family in particular includes the most recently acquired integrations, several of which are human specific and insertionally polymorphic (19). It also includes the most complete proviruses compared to any other family, leading to speculation of a naturally infectious HERV-K(HML-2) provirus. However, evidence for replication of a HERV-K(HML-2) virus in humans is still lacking. HERV-K(HML-2) proviruses appear unable to complete the replication cycle in humans and chimpanzees, but recent analysis of the gorilla genome identified HERV-K(HML-2) viruses that bear the hallmarks of very recent integration, raising the possibility of an infectious HERV-K(HML-2) virus still present in gorillas (20). Moreover, retrotransposition of a HERV-K(HML-2)-based reporter construct and mobilization of endogenous HERV-K(HML-2) have recently been reported in cells expressing the pluripotency transcription factor SOX2 in vitro (21). Thus, depending on the mouse strain, murine ERVs are able to complete the replication cycle in part or in full, and HERVs may be able to complete at least some of the steps.

Grouped with ERVs are also the mammalian apparent LTR retrotransposons (MaLRs), whose genomes are also flanked by LTRs (Figure 1). These, however, are nonautonomous ancient retro-transposons that lack the canonical retrovirus ORFs and have relied on ERVs for transposition (22). In addition to these LTR retroelements (ERVs and MaLRs), a larger proportion of the genome comprises EREs lacking LTRs (Figure 1). Phylogenetic analyses of the reverse transcriptase (RT) suggest common ancestry of LTR and non-LTR retroelements (9, 23). However, the replication cycles of these two types of ERE do exhibit notable differences. Long interspersed nuclear elements 1 (LINE-1) are a group of abundant non-LTR retroelements that contain autonomous retrotransposition-competent copies (24). These carry two functional ORFs, encoding an RNA-binding protein (ORF1p) and an enzyme with endonuclease and RT activities (ORF2p). Importantly, reverse transcription of LINE-1 RNA is primed by the integration target site and, therefore, takes place in the nucleus. Although ORF2p exhibits *cis* preference for LINE-1 mRNA binding, it is able to bind unrelated RNA transcribed from other EREs or cellular genes. Indeed, nonautonomous retrotransposition of other groups of non-LTR retroelements, including the prolific primate-specific *Alu* elements and other short interspersed nuclear elements (SINEs), and the hominid-specific composite SINE-VNTR-*Alu* (SVA) elements, relies on LINE-1 ORF2p (24).

The distinct life styles of LTR and non-LTR retroelements (Figure 1), together with their relative copy number and transcriptional patterns, can greatly influence the creation of ligands for innate and adaptive immune sensors and receptors and, hence, ERE immunogenicity.

## Endogenous Retroelements and Innate Immunity

All cellular organisms possess cell-intrinsic defenses against parasitism, but the evolution of multi-cellular organisms provided the opportunity to couple such cell-intrinsic immunity to organismal immunity through cellular communication. One prime example is the evolution of the interferon response system, initiated in some cells to warn other cells of infection (5, 6). Given the vast array of viruses and other parasites the immune system has to recognize and defend against, numerous sensors or receptors for different molecular patterns have evolved and are strategically placed in different subcellular locations (25–27). While some sensors have specificity for molecular patterns that are exclusively expressed by microbes, others recognize patterns that are also produced by the host but are usually shielded under physiological conditions (25–29). Consequently, discrimination between self-expressed and non-self-expressed ligands by the latter groups of sensors is not always absolute. Such discrimination may be particularly challenging for ligands expressed by EREs, which straddle host and virus.

Immune homeostasis would require the establishment of equilibrium between physiologically present ligands, including those potentially produced by EREs and tonic or regulated innate immune signaling. Disruption of this equilibrium by the introduction of foreign molecular patterns or dysregulation of self-expressed ligands would then trigger an innate immune response. As innate immune signaling cascades generally converge, particularly when type I interferon responses (referred to here as interferon responses) are elicited, it is often challenging to identify a single source or trigger for the response. Indeed, ligands from diverse sources can trigger the same sensor, and diverse sensors can elicit overlapping immune responses, such as transcription of interferon-stimulated genes (ISGs). Cases where specific ERE-expressed ligands uniquely initiate a signaling cascade, leading to a specific outcome, are the focus of intense investigation and are beginning to emerge (30, 31). However, much of the existing evidence points to a contribution of EREs to innate immune responses that may also be triggered by alternative self or foreign ligands, with the relative contribution of each source depending on the context (28, 29).

### Innate Immune Stimulation by Long Terminal Repeat Elements

**Figure 2:**
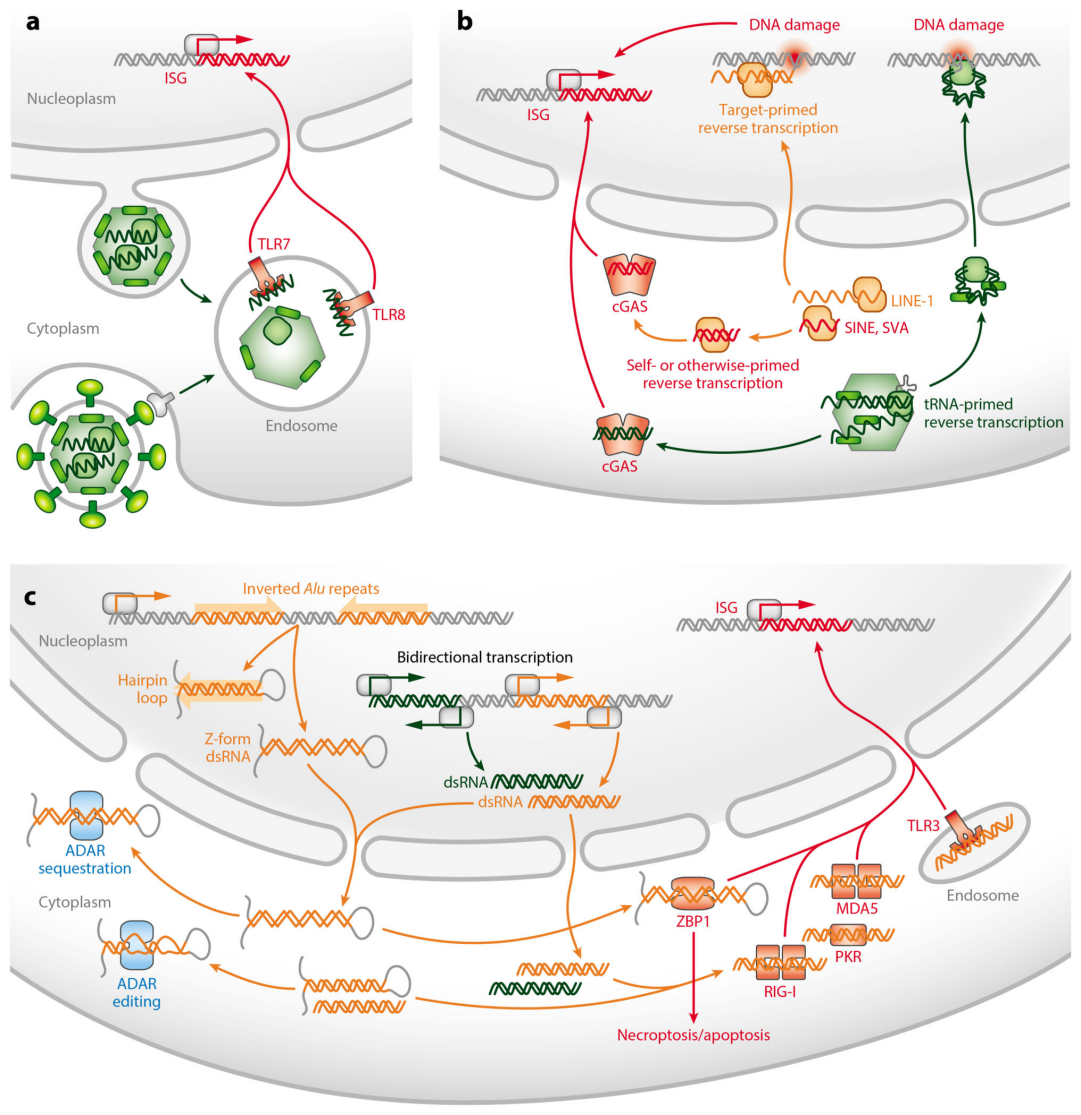
Immunogenicity of canonical and aberrant ERE nucleic acid replication intermediates. (*a*) Endosomal sensors of ssRNA, TLR7, and TLR8 may be triggered by ERV genomic RNA. This can derive from incoming extracellular virus particles accessing the endosome after cell entry or from intracellularly formed particles gaining access to the endosome through alternative routes, including autophagy (not depicted). Not depicted is the signaling cascade initiated by TLR7 and TLR8 ligation, which ultimately leads to the transcriptional induction of ISGs. (*b*) Cytoplasmic DNA sensors such as cGAS may be triggered by cDNA produced by ERVs, as part of the typical ERV replication cycle, or by non-LTR retroelements through aberrant cytoplasmic reverse transcription. The latter can be self-primed (as in the case of *Alu*) or primed by an as yet unknown mechanism. Triggering of cGAS and its downstream adaptor STING (not depicted) then leads to an ISG response. Separately, the endonuclease activities of ERV polymerase and LINE-1 ORF2p catalyze DNA breaks during the replication cycle, and the ensuing DNA damage may indirectly trigger an ISG response. (*c*) Potentially immunogenic dsRNA is also aberrantly produced by LTR and non-LTR retroelements through distinct mechanisms. Bidirectional transcription of EREs generates intermolecular complementary RNA, forming dsRNA. Transcription of inverted SINE/*Alu* repeats generates regions of intramolecular complementarity leading to the formation of hairpin loops. More enigmatic is the formation of Z-form dsRNA, also enriched for SINE/*Alu* sequences. The immunogenicity of hairpin loop and Z-form dsRNA may be reduced by ADAR-mediated editing and sequestration, respectively, but increased dsRNA formation or insufficient ADAR activity permits the triggering of several dsRNA sensors, including MDA5, RIG-I, PKR, and TLR3, initiating signaling cascades that converge to an ISG response. The Z-form dsRNA-binding protein ZPB1, which typically induces necroptosis or apoptosis, also contributes to the ISG response triggered when ADAR activity is reduced. Abbreviations: cDNA, complementary DNA; dsRNA, double-stranded RNA; ERE, endogenous retroelement; ERV, endogenous retrovirus; ISG, interferon-stimulated gene; LINE-1, long interspersed nuclear elements 1; LTR, long terminal repeat; SINE, short interspersed nuclear element; ssRNA, single-stranded RNA; SVA, SINE-VNTR-*Alu*; TLR, Toll-like receptor; VNTR, variable-number tandem repeat.

Similarly to exogenous retroviruses, individual steps in the typical replication cycle of ERVs create nucleic acid replication intermediates with the potential to engage multiple innate sensors (Figure 2). Single-stranded RNA (ssRNA) produced by ERV transcription is not inherently more immunogenic than other cytosolic RNA transcribed from cellular genes, but it may gain better access to the endosomal ssRNA sensors Toll-like receptor 7 (TLR7) and TLR8. The formation of transducing particles by complementation of defective ERVs provides the opportunity for such endosomal sensors to detect incoming ERV particles the same way incoming infectious viruses are detected. Moreover, for ERVs that have lost *env* and have adopted an intracellular life style, virus budding into the endoplasmic reticulum (ER) or endosomal vesicles may provide more direct access to endosomal sensors. More broadly, TLR7 is also accessible to cytoplasmic RNA through autophagy (32). Studies of genetic deficiencies in TLR7 or associated molecules in mice uncovered their critical role in the control of infectious recombinants derived from endogenous MLV (17, 33). However, ssRNA recognition likely targeted rescued recombinants more than defective endogenous MLV precursors in this context. Extracellular HERV-K(HML2) RNA has also been proposed as an endogenous ligand for TLR7 and TLR8 (34).

Reverse transcription of ERV genomes may also trigger innate sensors. Cytosolic DNA sensors lack sequence specificity and can be activated by multiple sources of DNA (26). Evidence supporting a role for reverse-transcribed DNA comes from studies of RT inhibitors in humans and animal models (30, 35–37), although the use of such inhibitors does not discriminate between ERV and LINE-1 RT, and it is more likely that these effects are mediated by LINE-1 RT, which, in principle, can reverse transcribe any polyadenylated RNA. Nevertheless, ERV RT has been specifically implicated in innate immune activation in some settings (38).

Unrelated to the typical retrovirus replication cycle, bidirectional expression of ERVs can generate complementary RNA that forms long double-stranded RNA (dsRNA), particularly in the context of natural or epigenetic drug-induced hypomethylation of cancer cells, which has been the subject of excellent reviews (31). Bidirectional ERV transcription may result from the bidirectional promoter activity of the proviral LTRs or from alternative adjacent promoters. Of note, dsRNA can form from certain IFN-γ-inducible loci carrying antisense ERV integrations in the 3′ untranslated region (UTR), pairing sense transcripts initiated by the STAT1-activated gene promoter and antisense transcripts initiated by the ERV LTR (39). ERV-derived dsRNA has been reported to activate TLR3 or MDA5 and downstream MAVS (40, 41). ERV-derived dsRNA produced by cancer cells has also been suggested to activate TLR3 in endothelial cells in mouse cancer models (42).

In addition to immunogenic nucleic acid replication intermediates, expression of canonical ERV proteins has also been associated with triggering of immune signaling or inflammation. Such activity may arise from the biological function of a particular ERV protein. For example, the Rec accessory protein of HERV-K(HML2) is a functional homolog of the Rev and Rex proteins of HIV-1 and human T cell leukemia virus I (HTLV-I), respectively, necessary for nuclear export of unspliced or partially spliced virus RNA (43). Rec-mediated export of potentially immunogenic RNA has been implicated in the transient innate immune activation during human embryo development, where ERVs are expressed as part of global epigenetic reprogramming (44).

The envelope glycoproteins of certain ERVs, including *Emv2* in mice and HERV-K(HML2) in humans, have recently been suggested to initiate signaling cascades in immune and cancer cells, when ligated with anti-envelope antibodies (45–48). Further dissection of the signaling pathways and their potential role in virus replication will help determine whether such activity is part of the physiological function of envelope glycoproteins. Similarly, overexpression of Syncytin-1, an exapted envelope glycoprotein (49), triggers inflammatory responses in human astrocytes in vitro and in transgenic mice in vivo (50), and overexpression of HERV-K(HML2) envelope glycoprotein triggers death in human neurons in vitro and in transgenic mice in vivo (51). An inflammatory response has also been reported in the brains of transgenic mice with genetic deletion of the epigenetic repressor *Trim28* in neural progenitor cells, which was linked with upregulation and accumulation of ERV-derived proteins, such as aggregates of intracisternal A particle (IAP) Gag proteins (52). Cell death has also been attributed to ERV protein accumulation following deletion of the epigenetic repressor *Setdb1* in pro-B cells (53). It remains unclear whether the reported toxicity of ERV proteins derives from their biological activities or their accumulation in sensitive cell types. Lastly, immunosuppressive activity has been described for a shared domain of several ERV envelope glycoproteins (49), but the underlying mechanism by which this activity may be exerted remains incompletely understood.

### Innate Immune Stimulation by Non-LTR Elements

Nucleic acid species and replication intermediates derived from non-LTR retrotransposons also possess immunogenic potential (Figure 2). Transfected *Alu* RNA has been reported to activate TLR7 and TLR8, but also to increase the immunogenicity of associated proteins in ribonucle-oprotein complexes (54, 55). Similarly to ERVs, bidirectional transcription of LINE-1 elements following loss of epigenetic regulators such as SETDB1 or components of the HUSH (human silencing hub) complex generates potentially immunogenic dsRNA in human cells (56, 57).

A connection between ERE derepression and the interferon response that follows treatment of cells with the epigenetic drug 5-aza-2′-deoxycytidine (5-Aza) has long been established. Leonova et al. (58) implicated dsRNA formation by SINE repeats, independently transcribed by polymerase III, in the induction of a suicidal interferon response triggered by 5-Aza treatment of murine primary cells, as well as cancer cell lines lacking p53. These findings suggested the existence of an alarm system monitoring the transcriptional activity of normally repressed EREs that, when triggered, leads to the induction of potent interferon responses. Subsequent studies with 5-Aza treatment of cancer cell lines (40, 41) or loss of the epigenetic repressor LSD1 (59) focused on ERVs as the potential dsRNA triggers of the interferon response. However, a major role for SINEs in the induction of the interferon response was recently highlighted by studies of the dsRNA-specific adenosine deaminase ADAR1, loss of which causes embryonically lethal interferon induction, dependent on recognition of self-dsRNA through MDA5 and its downstream adaptor MAVS (60–62). ADAR1 catalyzes adenosine-to-inosine (A-to-I) editing in dsRNA, thereby disrupting adenosine:uracil (A:U) base pairing and complementarity (63, 64). Investigation of ADAR1 targets revealed the dominant contribution of dsRNA structures formed by the transcription of inverted *Alu* repeats (63, 64). The presence of two or more *Alu* copies in reverse orientation in a single RNA transcript provides the sequence complementarity to form long hairpin loops that activate dsRNA sensors. Indeed, the lethal interferon response that develops in the absence of ADAR1 activity is thought to be driven by unedited dsRNA from inverted murine SINE (62) and human *Alu* repeats (65, 66), typically in the 3′ UTR of a limited number of genes. Moreover, thorough investigation of immunogenic dsRNA bound to MDA5 in 5-Aza-treated cancer cells identified inverted *Alu* repeats, and not ERVs as previously thought, as the main source (67). Under these conditions, intronic and intergenic inverted *Alu* repeats that are normally prevented from reaching the cytoplasm predominated the MDA5-bound fraction (67).

In addition to activating the MDA5-MAVS signaling cascade, self-dsRNA has recently been suggested to trigger inflammatory cell death through activation of ZBP1, a sensor of left-handed double-helical (Z-form) DNA and RNA (68–75). Where examined, the origin of Z-form dsRNA activating ZBP1 could be traced back to EREs. Indeed, activation of ZBP1 in mice with deficiency in necroptosis regulator RIPK1 or FADD (68) and in humans and mice with deficiency in SETDB1 (69) has implicated dsRNA from SINEs and ERVs, respectively. Moreover, several studies have now described an essential role for ZBP1 in the inflammatory cell death and fatal disease that develops when ADAR1 activity is reduced or when the Zα domain of its p150 isoform, responsible for Z-form dsRNA binding, is mutated (71–75). Mutation of the ADAR1 p150 Zα domain and, therefore, loss of binding to Z-form dsRNA do not appear to compromise the overall editing activity of ADAR1 (72, 73, 76). Although a small proportion of Z-form dsRNA, mostly derived from inverted SINE repeats, may be edited in a Zα domain–dependent manner (72, 76), differential interferon inducibility of ADAR1 isoforms may also produce such shifts in editing targets. These observations suggest that ADAR1 p150 may prevent ZBP1 activation by sequestering, rather than editing, Z-form dsRNA (72, 73, 76). The intersection of ADAR1 and ZBP1 pathways remains to be investigated, but it may involve physical interaction thought the Zα domains binding to the same Z-form dsRNA molecule, or competition for immunogenic SINE-derived Z-form dsRNA (71, 75). Of note, while ZBP1 activation has been primarily connected with the induction of various forms of cell death, including necroptosis, recent studies also revealed an indispensable contribution to the MAVS-dependent interferon response triggered when ADAR1 Z-form dsRNA binding is lost (73, 75).

Thus, Z-form dsRNA, or incompletely edited A-form dsRNA derived from SINEs, can trigger multiple signaling cascades converging on the interferon response. A central role for SINE-derived dsRNA in triggering an interferon response in multiple settings is further supported by the observation that expression of SINEs that can generate Z-form dsRNA or are targeted for A-to-I editing by ADAR1 is also induced by interferon (68, 72, 77). Moreover, their sensors, including ZBP1, as well as the Zα domain–bearing p150 isoform of ADAR1, are also interferon inducible in mice (71) and in human cancer (78), pointing to a finely regulated feed-forward loop of interferon induction.

Potentially immunogenic nucleic acids are additionally produced by the reverse transcription of LINE-1 and SINE RNA, a process that relies on LINE-1 ORF2p (79). A role for ORF2p-mediated reverse transcription was supported by findings in rare syndromes caused by dysregulation of nucleic acid metabolism or sensing, such as Aicardi-Goutières syndrome (AGS) (29). LINE-1 derepression and elevated ORF2p activity have been proposed to trigger the interferon response and inflammation that accompanies cellular senescence (80, 81). Furthermore, ORF2p activity is considered responsible for the interferon response that increases tumor immunogenicity in mice and humans, an enhanced form of which has been described for the cancer-resistant blind mole rat (82).

To prevent recognition of nuclear DNA, the activity of DNA sensors, such as cGAS and its downstream adaptor STING, is typically restricted to the cytoplasm (26). Although target-primed reverse transcription mediated by ORF2p is restricted to the nucleus, recent evidence suggests that LINE-1 and SINE complementary DNA (cDNA) can also be made in the cytoplasm. Indeed, LINE-1 cDNA and self-primed *Alu* cDNA have been detected in the cytoplasmic fraction of murine or human cells (80, 83), providing a possible mechanism for triggering the interferon response.

Lastly, the fully functional ORF2p, as well as numerous ORF2p copies that retain only the endonuclease activity, can introduce DNA double-strand breaks, triggering a DNA damage response (84). In turn, genomic DNA damage can initiate the cGAS-STING signaling cascade, leading to an interferon response (85), thus providing an alternative means of interferon activation by ORF2p.

## Endogenous Retroelements and Adaptive Immunity

The combinatorial process of T cell receptor (TCR) and B cell receptor (BCR) gene segment rearrangement produces random specificities, some of which may be directed against self-antigens. Consequently, powerful mechanisms including negative selection of potentially autoreactive antigen receptors; naturally suppressive cells, such as regulatory T cells (Tregs); and inhibitory ligand/receptor axes, such as PD-L1/PD-1, have evolved to ensure immunological tolerance to self-antigens. Nevertheless, autoreactive TCRs and BCRs do develop and do occasionally break through regulatory and inhibitory controls to cause autoimmunity.

As part of self, proteins encoded by EREs should also be immunologically tolerated by the adaptive immune repertoire. However, specific features of ERE proteins may substantially modify their immunogenic or tolerogenic activity.

Firstly, certain endogenous retroviral envelope glycoproteins exhibit long-recognized super-antigen activity for reactive TCR Vβ families in mice and humans (86). These include several endogenous MMTV proviruses in mice (87) and a HERV-K18 provirus in the first intron of the *CD48* gene in humans (88). Superantigen reactivity may be particularly enriched in Tregs, which typically escape negative selection by self-antigens, including retroviral superantigens (89). Indeed, in the commonly used C57BL/6 strain of laboratory mice, 10% of all Tregs are reactive with an endogenous MMTV superantigen (90), and their function can be directly modulated by changes in endogenous MMTV superantigen expression during inflammation or unrelated infection (91, 92).

Secondly, not all ERE-encoded proteins are or need to be expressed under physiological conditions. In contrast to all other host proteins, which are evolutionarily selected for a given function, most ERE protein products are unlikely to have retained their original function or assumed new function. Their expression is not, therefore, subject to positive selection, and it may even be actively suppressed as part of the epigenetic control that the host imposes on the transcription of many EREs. Insufficient expression of such EREs under physiological conditions also leads to incomplete immunological tolerance of their products and instead may create immunological ignorance. However, failure of these control mechanisms, as it occurs in cancer or in autoimmunity, may also lead to immune reactivity against these otherwise ignored antigens.

A third feature of certain ERE-encoded proteins that may enhance their immunogenicity is their assembly into inherently immunogenic structures, such as virus particles, capsids, and ribonucleoprotein complexes. Formation of transducing particles through complementation between defective proviruses, such as endogenous MLVs, and even restoration of infectivity through recombination, giving rise to fully infectious retroviruses (12), is greatly facilitated by better overall conservation of ERVs in laboratory mice than in humans. Nonetheless, formation of retrovirus particles by human ERVs has also long been suspected or directly observed. Indeed, retrovirus particles have been detected in normal human placentas (93–95), attributed to HERV-K proviruses (96). Similarly, retrovirus particles produced by human teratocarcinoma cell lines were traced to HERV-K proviruses (97). HERV-K(HML-2)-derived particles are present in the plasma of lymphoma patients and can mobilize HERV-K-related proviruses (98, 99). Although it remains unclear whether other human ERVs can produce particles, the formation of particles by at least some HERV-K proviruses would enhance the immunogenicity of HERV-K proteins, as well as proteins from other ERVs that may be incorporated into HERV-K particles.

Lastly, comparison of the HLA class I–presented epitopes derived from ERVs with those from other human proteins or exogenous viruses infecting humans revealed unique features of ERV-derived epitopes that could potentially enhance their immunogenicity (100). ERV epitopes showed higher sequence identity with virus than human protein epitopes and enrichment for amino acid residues in specific epitope positions (100).

### Adaptive Immune Responses to Endogenous Retrovirus Proteins in Humans

While the contribution to immunogenicity of unique features of ERE-encoded proteins remains to be quantified, spontaneous adaptive immune responses against such proteins have been consistently reported both in mice and in humans. The most frequent targets of adaptive immune responses appear to be canonical ERV proteins, likely owing to better or earlier annotation of these proteins, examples of which are listed below.

#### ERV3–1 envelope

One of the earliest demonstrations of adaptive immune reactivity to an ERV-encoded protein in humans is that against the envelope glycoprotein of the *ERV3–1* provirus. A member of the HERV3 family (also known as HERV-R), *ERV3–1* is a single-copy provirus on chromosome 7q11.21 and one of the best-studied ERVs (101, 102). It is present only in old-world primates, except gorillas, and the human copy has sustained mutations in all ORFs expect the *env* ORF (101, 102). The *ERV3–1* envelope glycoprotein is expressed in normal syncytiotrophoblasts and had been speculated to function similarly to syncytins, endogenous retroviral envelope glycoproteins that are essential for syncytiotrophoblast formation during placentation (49). However, despite its preservation, the *ERV3–1 env* ORF carries a polymorphic premature stop codon in ~1% of White people that does not compromise successful pregnancy (103, 104).

In early studies, sera from pregnant women, as well as those from patients with Sjögren syndrome or systemic lupus erythematosus (SLE), exhibited increased antibody reactivity against a peptide corresponding to a predicted epitope from the *ERV3*–*1* envelope glycoprotein and to recombinant protein (105). The highest antibody levels were found in the sera from mothers of infants with congenital heart block, a rare pregnancy-associated autoimmune disorder (105). However, in a subsequent study of 12 mothers of infants with congenital heart block, none was homozygous for the *ERV3–1 env* allele with the premature stop codon (103), arguing against a mechanism whereby lack of immunological tolerance due to lack of *ERV3–1* envelope expression in mothers permits the induction of a pathogenic response against this glycoprotein expressed in the fetus as a paternal alloantigen.

More recently, IgG antibodies reacting with the full-length *ERV3*–*1* envelope glycoprotein expressed on the target cell surface have been detected in sera from a fraction of juvenile-onset SLE patients, as well as patients with multisystem inflammatory syndrome in children (MIS-C) following SARS-CoV-2 infection (106). Collectively, these studies highlight a possible association of *ERV3–1* envelope antibodies with autoimmune manifestations.

#### HERV-K(HML-2)

Arguably, the most frequently targeted canonical ERV-encoded proteins in humans belong to the HERV-K(HML-2) family, which includes the most recent and most complete proviruses (19). Consequently, numerous HERV-K(HML-2) proviruses in the human genome carry intact and highly similar *gag, pol*, and *env* ORFs (19). In turn, it is often difficult to establish which particular HERV-K(HML-2) copy might have induced a T cell or B cell response that cross-reacts with the products of other similar copies. For example, of the 14 HERV-K(HML-2) proviruses with a fully or partially intact *env* ORF in the human genome, at least 8 encode products with 95–98% amino acid identity between them and 98–99% amino acid identity with the HERV-K(HML-2) envelope glycoprotein consensus.

T cell responses to HERV-K(HML-2) proteins have been detected primarily in the context of cancer, targeting epitopes encoded by *gag* or *pol* ORFs (107, 108). Antibody responses to HERV-K(HML-2) envelope glycoprotein and Gag precursor protein have also been frequently observed in several cancer indications (109–117). Notably, antibodies reactive with HERV-K particle– producing teratocarcinoma cells were also detected, albeit at low titers, in the sera of a small proportion (~4%) of healthy individuals and of pregnant women (117), suggesting that spontaneous autoreactivity against HERV-K(HML-2) envelope glycoproteins can arise in individuals without overt pathological manifestations. Antibodies, primarily of the IgM class, to recombinant HERV-K(HML-2) envelope glycoprotein and Gag precursor protein were also detected by ELISA in an independent study of healthy individuals and were reported to be reduced in psoriasis patients (118).

Antibody responses to recombinant HERV-K(HML-2) envelope glycoprotein have also been reported in a recent study of healthy individuals and SLE patients (119). These were primarily of IgG subclasses and were found at comparable titers between healthy donors and SLE patients. In this study, Tokuyama et al. (119) were able to pinpoint a particular HERV-K(HML-2) provirus on chromosome 1q22, *ERVK-7* (also known as *HERV-K102)*, as the most likely source of the targeted envelope glycoprotein. This provirus exhibited the highest expression among all HERV-K(HML-2) proviruses examined both in healthy donors and in SLE patients and was also upregulated in SLE patients. This study suggests that, similarly to *ERV3–1*, the product of a single HERV-K(HML-2) provirus alone may be immunogenic and responsible for the observed immune reactivity.

Titers of antibodies reacting with the consensus HERV-K(HML-2) envelope glycoprotein expressed on the cell surface were also comparably low in children and adolescents with autoimmune rheumatic diseases and age-matched controls, but substantially higher in MIS-C patients (106), indicating that such antibodies may be induced in the weeks following acute viral infection, such as with SARS-CoV-2, but not necessarily associated with autoimmune rheumatic diseases.

#### HERV-E

Members of the HERV-E family have been found to be highly immunogenic particularly in clear cell renal cell carcinoma (ccRCC). Graft-versus-tumor effect in a ccRCC patient who underwent hematopoietic stem cell transplantation led to the identification of a HERV-E provirus on chromosome 6q15 (also known as CT-RCC) as the source of the tumor-associated epitope targeted by donor CD8^+^ T cells (120). This provirus shows minimal expression in healthy tissues, including the kidney, but is strongly upregulated in ccRCC, likely through the combined effects of epigenetic derepression, inactivation of the *VHL* tumor suppressor gene, and overexpression of the HERV-E LTR transcription factor HIF-2α (121). As a result, translation products from partial *gag-pro-pol* and *env* ORFs are produced specifically in ccRCC, triggering T cell responses (120, 122).

A more comprehensive search of potentially immunogenic HERVs in ccRCC additionally identified another HERV-E family member on chromosome 19q12, also expressed specifically in this indication, with products from partial *gag-pro-pol* ORFs targeted by a substantial proportion of tumor-reactive CD8^+^ T cells (123). The repeated identification of HERV-E family members as the triggers of adaptive immune responses in ccRCC underlines the commonalities in their regulation during development of this cancer type.

#### HERV-H

The HERV-H family is a large grouping of diverse proviruses with some unusual characteristics. Most proviruses were acquired before the split of old-world and new-world monkeys about 40 million years ago. Some members were acquired even earlier than that, whereas other members are specific to great apes (19). Despite their age, an atypically larger proportion of HERV-H proviruses have retained proviral sequences between the two LTRs, in contrast to most other families, whose members are predominantly in solo LTR form (124). The ORFs in the retained internal sequences are not necessarily intact, and indeed, the *env* genes of HERV-H family members show a decay rate that is typical of all ERVs and are highly mutated (124). Nevertheless, numerous HERV-H family members are expressed in healthy cells and may be linked with physiological processes, most notably transcriptional regulation of human stem cell pluripotency (125). Expression of HERV-H proviruses in healthy cells does not, however, negate the immunogenicity of all copies in the genome, and CD8^+^ T cell responses have been documented against products of one particular HERV-H provirus on chromosome Xp22.3 (126). This provirus is strongly upregulated in gastrointestinal cancers, and in addition to a partial *gag* ORF, it contains an atypical ORF upstream of the *pre-gag* ORF present in certain other retroviruses (127). The product of this atypical retroviral ORF, rather than the reported *env* product (126), was the likely target recognized by CD8^+^ T cells in colorectal cancer cell lines.

#### HERV-W

In multiple sclerosis patients, elevated antibody reactivity has also been reported against peptides corresponding to the envelope glycoproteins of HERV-W proviruses (128, 129), including Syncytin-1, encoded by the *ERVW-1* provirus on chromosome 7q21.2 (49), and multiple sclerosis–associated retrovirus (MSRV), a speculated but so far elusive virus thought to be related to HERV-W proviruses (130). However, reactivity against insect cell–produced, full-length versions of the envelope glycoproteins revealed no reactivity against the putative MSRV envelope glycoprotein and very rare reactivity against Syncytin-1 (131).

#### Additional and unconventional ERE-encoded antigens

Recent technical advances in protein identification through mass spectrometry, as well as in detection of reactive T cells, have allowed more comprehensive discovery of putative antigens encoded by ERVs and other retroelements, particularly in the context of cancer.

Using a limited list of 66 annotated HERVs in conjunction with DNA barcode–labeled MHC-I multimers, Saini et al. (132) identified CD8^+^ T cells reactive with numerous peptides encoded by canonical retroviral ORFs in members of the HERV-K, HERV-H, HERV-W, and HERV-E families in patients with hematological cancers. CD8^+^ T cells reactive with some of these peptides were also found in healthy donors, albeit less frequently, suggesting clonal expansion in cancer patients (132). In an analysis of all annotated genomic EREs, two studies identified ORFs within LTR retroelements, as well as LINE-1, SINEs, and composite SVA elements, as the source of peptides presented by MHC-I in glioblastoma (133, 134). Antibodies reactive with LINE-1 ORF1p have been detected in most SLE patients, as well as in a smaller proportion of healthy individuals, and their titers were higher in active SLE (135).

The identification of peptides derived from the translation of ERE transcripts not previously thought to have coding potential is consistent with accumulating evidence for translation of unconventional ORFs, including 5′ and 3′ UTRs, pseudogenes, long noncoding RNAs, short ORFs, and alternative ORFs (136–142). However, in addition to unconventional ORFs present in a given RNA molecule, alternative splicing patterns and use of alternative start or polyadenylation sites can create diverse RNA isoforms transcribed from a given locus, carrying additional putative ORFs. Indeed, increased alternative splicing in most human cancer types may create novel peptides that include predicted MHC-I binders (143). Moreover, increased intron retention in ovarian cancer (144), acute myeloid leukemia (145), and SF3B1-mutated uveal melanoma (146) has been implicated in the creation of tumor-specific T cell epitopes.

**Figure 3:**
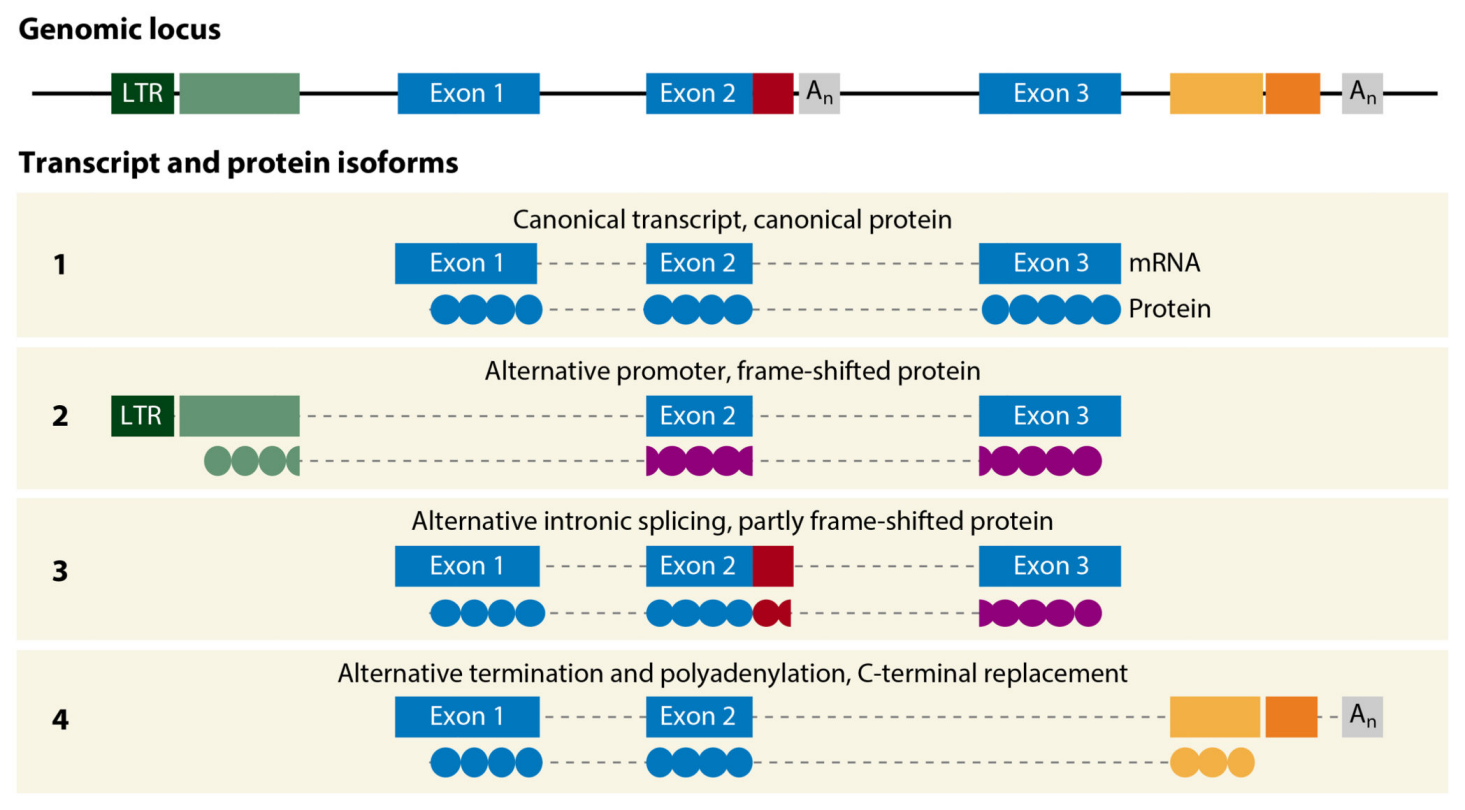
Generation of aberrant protein products by transcriptional utilization of EREs. A hypothetical example of a protein-coding gene consisting of three exons with EREs integrated within and around the gene body (*top*) and potential alternative splicing isoforms of transcribed RNA (*bottom*). Isoform 1 represents the canonical mRNA and translated protein. Isoform 2 uses an LTR element as an alternative promoter, skipping the first canonical exon and shifting the translation frame. Isoform 3 uses an alternative splice donor site in an intronic SINE element instead of the canonical site at the end of exon 2, shifting the remaining translation. Isoform 4 uses a LINE-1 fragment as alternative terminal exon, replacing the C-terminal sequence of the translation product. Abbreviations: ERE, endogenous retroelement; LINE-1, long interspersed nuclear elements 1; LTR, long terminal repeat; SINE, short interspersed nuclear element.

Given their genomic structure and sheer copy numbers, EREs represent the largest source of alternative start, polyadenylation, and splicing sites in the genome (7, 8). Their transcriptional utilization, therefore, has the potential to increase RNA isoform diversity quite considerably (Figure 3). Transcripts originating from or including EREs are still incompletely annotated, but recent efforts using de novo transcriptome assemblies without bias against repetitive sequences have uncovered increased ERE utilization in novel transcripts, often in a cancer type–specific manner (147, 149). Inclusion of EREs in chimeric transcripts also overlapping unique neighboring genomic regions creates the potential for translation of protein products (Figure 3), with far less homology with products from other parts of the genome than products translated from repetitive EREs alone and canonical retroviral ORFs that may also be expressed in healthy tissues (100, 147). Indeed, recent studies integrating improved annotation of ERE-overlapping transcripts with immunopeptidomic analyses highlighted the considerable contribution of such chimeric products to the antigenic identity of cancer cells (147–150, 151). Moreover, the overlap of antigenic transcripts identified in each study using different methodologies is partial, suggesting that the complete contribution of chimeric ERE-overlapping transcripts to cancer antigenicity has not been captured yet.

#### Adaptive Immune Responses to Endogenous Retrovirus Proteins in Animal Models

Despite the continuously evolving relationship between ERVs and the immune system of the host, ERV products can be comparably immunogenic in humans and in animal models, but there are also notable differences. Spontaneous B cell responses to ERV products have long been detected in mice, particularly from autoimmune-prone strains, where they have been implicated in SLE development (152). These autoantibody responses were found to target predominantly the envelope glycoprotein of endogenous MLVs (152). Similarly, B and T cell responses are known to be induced against endogenous MLVs expressed in transplantable cell lines used in murine cancer models (153–156). In the commonly used C57BL/6 mice, these adaptive immune responses target most frequently the envelope glycoprotein of *Emv2*, a single-copy endogenous MLV that induces partial immunological tolerance (157). Although *Emv2* is a replication-defective provirus, recombination with other defective endogenous MLVs can restore its infectivity, resulting in fully infectious, now exogenous retrovirus. Restoration of *Emv2* infectivity was first described in B16 melanoma cells, where it gave rise to the melanoma-associated retrovirus (MelARV) that reinfected these cells multiple times (15, 158). Notably, it was the hunt for the immunodominant target of anti-B16 melanoma antibodies that ultimately lead to the discovery of MelARV (153, 154), suggesting that endogenous MLVs are major cancer antigens. Restoration of infectivity likely contributes significantly to the heightened immunogenicity of endogenous MLVs and has been reported in most transplantable cell lines (16, 18).

Restoration of *Emv2* infectivity, leading to vertical transmission of infectious MLVs, has also been documented in mouse strains with genetic deficiencies affecting antiretrovirus antibody production (17, 33), underscoring the notion that adaptive immune responses to endogenous MLVs are not only possible to induce but are also necessary to control the emergence and transmission of infectious recombinants.

In addition to MLVs, adaptive immune responses against the envelope glycoproteins of endogenous MMTVs have also been documented. For example, T cell responses to EL4 T cell lymphoma cells have long been shown to target epitopes from MMTV envelope glycoprotein (159). Similarly to MLVs, MMTVs can also exist as defective endogenous and infectious exogenous viruses in certain mouse strains, and they have been suggested to amplify their copies in transplantable murine lymphoma cell lines (160). More recent studies, including those using high-throughput discovery of cancer antigens, have independently identified epitopes from canonical MLV and MMTV proteins that reinforce their immunogenicity. In a comprehensive study of tumor-specific antigens by Laumont et al. (150), the most immunogenic and protective epitope identified in EL4 cells was, in fact, derived from the MMTV envelope glycoprotein. The envelope glycoprotein of *Emv2* has also been validated as tumor antigen in the transplantable GL261 glioma model (161). Moreover, a genetic screen of chromatin regulators that modify immunogenicity of mouse cancer models identified derepressed endogenous retroviruses as the predominant targets in *Setdb1*-deficient cancer cells, with enrichment for peptides from *Emv2* envelope glycoprotein, as well as endogenous MLV Gag and polymerase (162). The recurrent identification of endogenous MLV and MMTV antigens in independent studies points to a major contribution to mouse cancer cell immunogenicity, likely accentuated by the increase in MLV and MMTV copy numbers in transplantable cell lines.

## Consequences Of Host Immune Responses To Endogenous Retroelements

**Figure 4:**
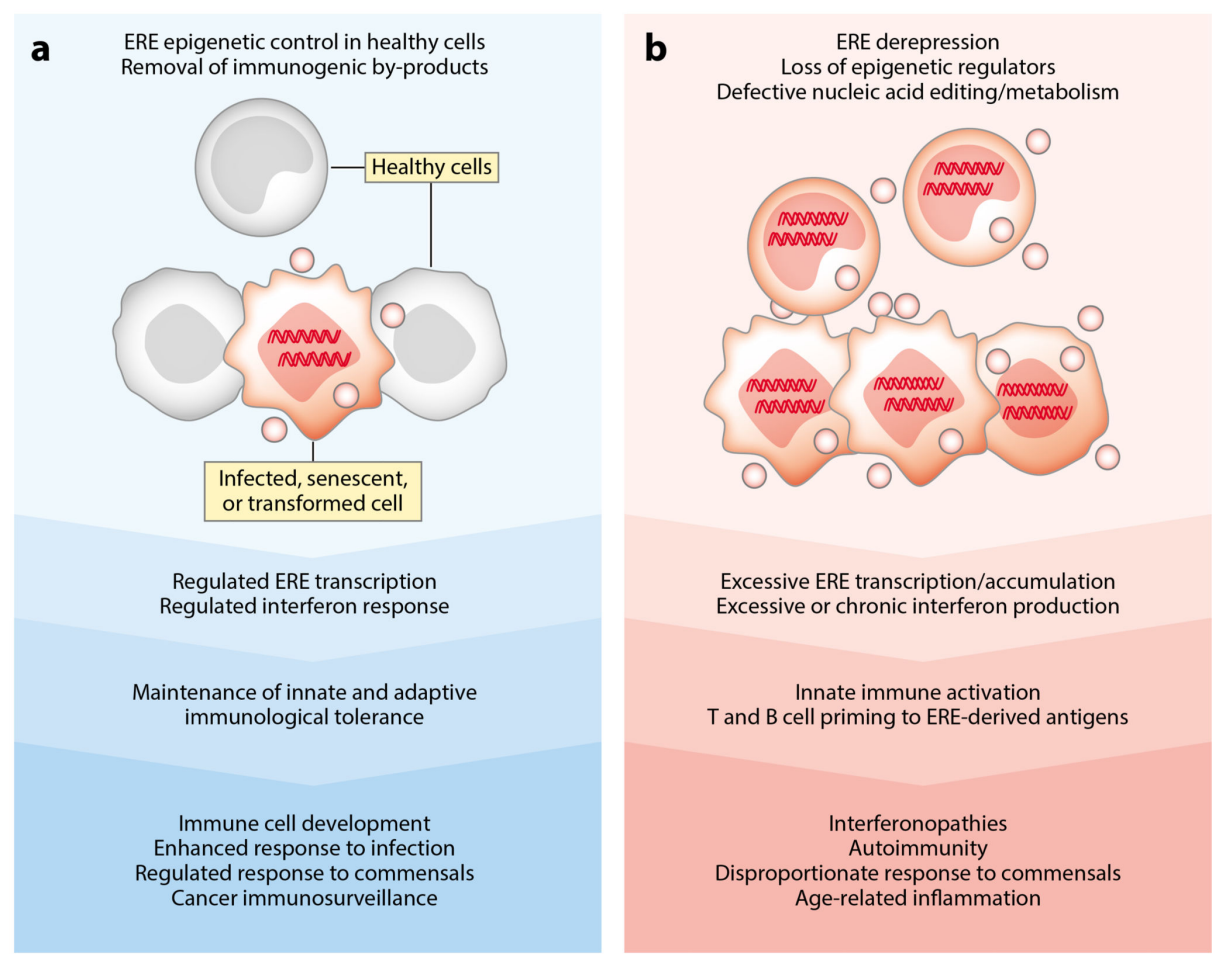
Proposed consequences of ERE immunogenicity, depending on the degree of ERE activity. In physiological conditions (*a*), ERE transcriptional activity is epigenetically controlled and their products eliminated in most cells, with the exception of infected, transformed, senescent, or otherwise stressed cells. In turn, this prevents interferon responses and priming of adaptive immune cells against the low level of ERE products in healthy cells but permits immune reactions against elevated ERE products in stressed cells. Such regulated responses are thought to contribute to several physiological processes. In contrast, when ERE transcription is unleashed (*b*), through loss of epigenetic control, or their products accumulate, through loss of nucleic acid metabolism or editing machineries, in a sufficient number of otherwise healthy cells, the resulting excessive interferon and adaptive immune responses can trigger or contribute to a range of pathological conditions. Abbreviation: ERE, endogenous retroelement.

As EREs are closely intertwined with the host, immune reactivity against their products could be considered autoimmune in nature and, therefore, has the potential to cause pathology (Figure 4). A pathogenic contribution of ERE-triggered interferon responses is supported by findings in AGS and SLE, as well as in atrophic macular degeneration and age-related inflammation (29, 30, 37, 83). The nucleic acid sensors or metabolic enzymes implicated in the development of these conditions lack the specificity that could discriminate between ERE-derived and other self–nucleic acids (25, 26, 29). Nevertheless, a causative role for EREs is indicated by the use of RT inhibitors. These have shown efficacy in early human AGS trials (37), although results from mouse models of AGS have been conflicting (36, 163). A potential effect of RT inhibitors on SLE would also be important to establish. RT inhibitors also reduce inflammation in aged mice (80), and inspection of health insurance databases indicates that RT inhibitors given as part of pre-exposure prophylaxis to individuals not infected with HIV-1 may lower the risk of atrophic macular degeneration and type 2 diabetes (83, 164). However, proinflammatory effects of pre-exposure prophylaxis have also been reported in genital or gastrointestinal mucosae following topical application of RT inhibitors (165, 166), and in the gastrointestinal mucosa but not the blood following oral administration in individuals not infected with HIV-1 (167). Proinflammatory effects have also been reported in colorectal cancer patients treated with RT inhibitors and in similarly treated colorectal cancer cell lines, particularly those with p53 mutations (168), which are hypothesized to arise from residual ORF2p activity and lead to DNA damage or accumulation of DNA replication intermediates. Collectively, these findings underscore a nonredundant pathogenic potential of RT encoded by either LINE-1 or ERVs, which warrants further investigation into its source and regulation.

A pathogenic contribution of dysregulated innate immunity to EREs does not, however, preclude a beneficial role in physiological conditions (Figure 4). Indeed, innate immune activation by EREs has been suggested to augment the interferon response to exogenous, unrelated viruses, such as herpesviruses (169) or influenza A virus (170), although these viruses can also subvert or inhibit ERE-induced interferon responses. ERE-triggered proinflammatory signaling downstream of MDA5 and RIG-I is thought to be required for hematopoietic stem and progenitor cell development (171). Activation of the cGAS-STING pathway by EREs has also been suggested to play a major role in promoting the homeostatic adaptive immune response to skin microbiota (38). Therefore, the induction of ERE transcription during infection or colonization with exogenous microbes may function as an intrinsic adjuvant necessary for tuning innate immune reactivity. A beneficial effect of EREs in immune defense from exogenous pathogens has also been described in a mouse model for infection with herpes simplex virus type 2, where the effect was independent of type I interferon signaling (172), suggesting additional mechanisms by which this interaction can manifest at epithelial barriers. Moreover, a large body of evidence suggests a host-protective role for EREs against cancer initiation and progression. Indeed, transcriptional upregulation of EREs in response to mutation of key epigenetic regulators, oncogene activation, cytokines, or natural and drug-induced genomic hypomethylation is associated with potent antitumor effects, attributed to innate immunity induced intrinsically in cancer cells, and increased tumor immunogenicity (39–41, 56, 59, 77, 78, 123, 162, 173–181), although ERE-induced genome instability has also been reported (182).

The link between ERE transcriptional activity and cell-intrinsic innate immune activation may provide tonic and amplifying signals for the innate immune system, as well as the means of effective immunosurveillance of cells damaged by stress, infection, mutation, or transformation (183, 184). Disruption of such physiological processes may, in turn, explain the pathogenic consequences of ERE activation. For example, excessive ERE activation promoted by a high-fat diet can turn homeostatic responses to the microbiota into inflammatory responses (38). Similarly, age-related inflammation, SLE, and AGS may represent the spectrum of ERE dysregulation. Lastly, ERE upregulation in cancer may also be detrimental, particularly when the interferon response pathways are disrupted (180, 185), which may also explain increased cancer susceptibility in old age, despite increased ERE activity (82).

A pathogenic potential of adaptive immune responses to EREs can also be envisioned. Although their titers were not elevated, antibodies reactive with HERV-K(HML-2) envelope glycoproteins were found to cause neutrophil activation in SLE patients (119). Antibodies to HERV and LINE-1 products have frequently been observed in autoimmune disorders, but also in otherwise healthy individuals, albeit often less frequently (106, 135, 172). Consequently, their contribution to disease initiation and progression remains unclear (186). Germline ERVs, as well as maternally transmitted infectious counterparts, induce a degree of T cell tolerance in mouse models (157, 187). However, B cells appear to escape deletional tolerance and ultimately mount an anti-ERV antibody response in the offspring that protects further vertical transmission of infectious viruses, without pathological signs (187). Moreover, induction of adaptive immune responses against LINE-1 ORF2p and HERV-K(HML-2) Gag and envelope proteins by vaccination was not associated with adverse or pathological findings (188). Adaptive immune responses against ERV-encoded antigens can afford protection against cancer, both in mouse models and in humans (189). However, whether such responses are additionally associated with paraneoplastic autoimmunity or adverse effects of checkpoint blockade remains an important question.

## Concluding Remarks And Broader Perspective

While both innate and adaptive responses to diverse ERE products can be clearly elicited, the overall consequences for the host are context dependent. For a powerful defense weapon such as the adaptive immune system, its response to a given stimulus may range from host protection to immune pathology or autoimmunity (190), and responses to EREs seem to follow the same rules. Equally, innate immune activation by EREs may contribute to protection from cancer but also promote age-related inflammation (30). Such antagonistic pleiotropy has striking parallels with dichotomous regulation of cancer risk and lifespan by p53 (191), or cancer risk and regenerative ability (192).

Participation of EREs in multiple biological processes implies that evolutionary adaptation will be slower, but persistence in the host will be more likely. The adaptation of EREs and host immunity will naturally integrate stronger evolutionary pressure on the host immune system from a multitude of exogenous pathogens, as well as ERE effects on host biological processes other than the immune system. Fitness trade-offs associated with ERE activity in the host can manifest at many levels, but arguably, a major battleground is genome function (7, 8, 79, 183, 193). Indeed, genomic features of EREs, together with their abundance, provide the genetic diversity for evolution of new gene function, including of immune genes.

A growing list of examples comprises the interferon inducibility provided by *MER41* LTR elements to several genes, including the cytoplasmic DNA sensor *AIM2* (194), and the enhancer activity provided by an *ERV1* LTR promoting HLA-G expression in human extravillous trophoblasts at the fetal-maternal interface (195). ERE-driven innate immune activation of dendritic cells lacking TRIM28 is linked to transcriptional regulation of nearby immune genes, rather than ligation of innate sensors (196), and LINE-1-containing transcript isoforms of immune genes regulate naive T cell quiescence (197). Alternative splicing events exonizing EREs can create isoforms of immune gene transcripts with novel function, such as the soluble form of PD-L1 acting as a receptor antagonist (198), and polymorphic *Alu* integrations in intronic regions can cause skipping of nearby exons, affecting the function of immune genes such as *CD58*, which encodes lymphocyte function–associated antigen 3 (LFA-3) (199).

While major effects of EREs in immune gene function are likely selected over long evolutionary periods, the regulation of ERE activity, particularly in healthy cells, and the potential effects of EREs on gene function are largely unexplored. Great progress is being made in defining ERE-derived ligands and respective innate sensors and adaptive receptors, triggering of which leads to immune activation in health and disease. However, our understanding of the full interaction of EREs with the host immune system will only be completed with integration of their roles as immune gene regulators, as well as immune activators.

## Footnotes

1 The term viral mimicry is used here to describe a virus strategy whereby virus components molecularly or functionally resemble respective host components. In its simplest form, this strategy is analogous to those employed by many organisms that avoid detection by blending into the background, such as the octopus mimicking the seabed, as Aristotle noted. The term is also used elsewhere in the literature to describe the reverse phenomenon, whereby the host produces molecular patterns that resemble those usually produced during virus infection and that induce an antiviral state, characterized by a type I interferon response. Where interferon responses are triggered by endogenous retroviruses—which are, rather than mimic, viruses—we refer to this phenomenon simply as induction of interferon responses.
